# To What Extent is Anfinsen’s Thermodynamic Hypothesis Consistent With the Formation and Polymorphism of Amyloid Fibrils? ^☆^

**DOI:** 10.1016/j.jmb.2025.169364

**Published:** 2025-07-30

**Authors:** Yi Xiao Jiang, David S. Eisenberg

**Affiliations:** Department of Biological Chemistry, Department of Chemistry & Biochemistry, UCLA-DOE Institute, and Molecular Biology Institute, UCLA, Los Angeles, CA 90095, USA

**Keywords:** Anfinsen’s thermodynamic hypothesis, amyloid fibrils, amyloid nucleation, structural polymorphism, intrinsically disordered proteins

## Abstract

For half a century, Anfinsen’s Thermodynamic Hypothesis has been considered the central pillar of protein science. In Anfinsen’s words, this hypothesis holds that “…the three-dimensional structure of a native protein in its normal physiological milieu is…the one in which the Gibbs free energy of the whole system is lowest; that is, that the native conformation is determined by the totality of interatomic interactions and hence by the amino acid sequence, in a given environment”. Applying this hypothesis to amyloid fibril-forming proteins presents challenges, which we contemplate in four questions. First, what is the “native” structure of amyloid-forming proteins, many of which are intrinsically disordered or are proteolytic fragments of larger proteins? Second, what is the thermodynamic landscape for the conversion of native monomers to highly stable fibril assemblies? Third, how do we reconcile Anfinsen’s hypothesis, that a protein’s amino acid sequence determines its 3-dimensional structure, with amyloid fibrils, for which single protein sequences are capable of folding into multiple polymorphs? Fourth, what is the “physiological milieu” of amyloid fibrils? Is it increased local concentration, cofactor binding, post-translational modifications, or cellular programming of diseased tissues? We discuss answers supplied by *ex vivo* observations and *in vitro* experiments, and conclude that amyloid protein structure *in vivo* is determined by its sequence and its physiological milieu.

## Applying Anfinsens hypothesis to amyloid fibrils

Modern biology has been conceptually shaped by Anfinsen’s thermodynamic hypothesis, which states that a protein’s structure is its free energy minimum conformation in its environment, determined by its amino acid sequence, coded by its gene sequence [1]. Of the ~20,000 proteins in the human proteome, most are globular or membrane-associated [2], and form structures that appear consistent with this model. Amyloid fibrils, the hallmarks of numerous neurodegenerative and systemic diseases, are built by many copies of the same protein in β strand layers. Properties of amyloid fibrils, including single sequences that give rise to multiple structures (polymorphism), unusual stability that resists disassembly, and intrinsically disordered domains have led to considerations that amyloid fibrils may be inconsistent with Anfinsen’s dogma [3-7]. Here, we examine the formation and polymorphism of amyloid fibrils through the lens of the thermodynamics, to explore the extent to which Anfinsen’s hypothesis can be applied. We limited the scope our review to exclude some topics under vigorous investigation, such as the evolution of functional amyloids, as well as oligomers and intermediate states of aggregation.

## Native structure of amyloid-forming proteins

**Figure 1.**
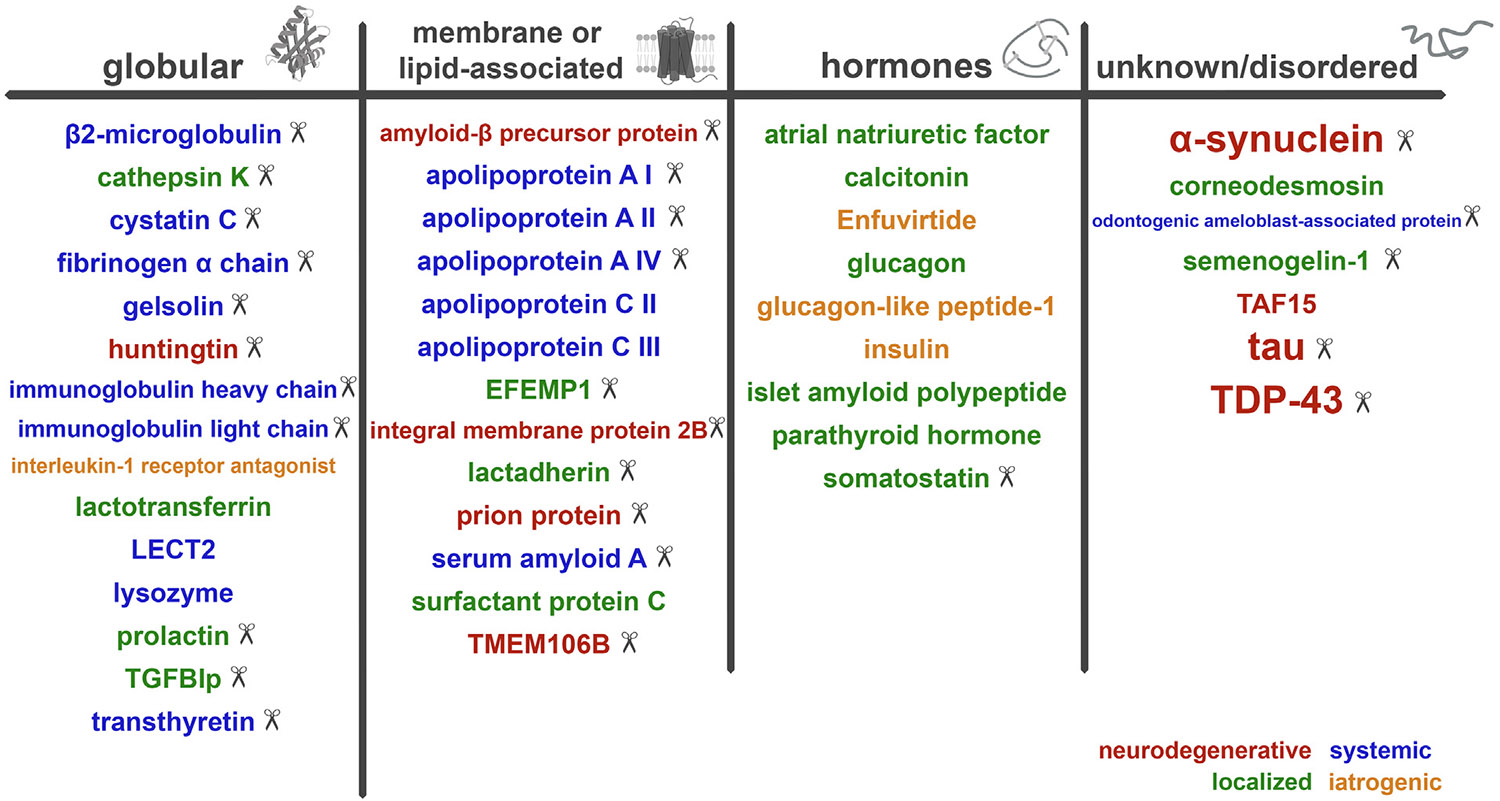
Native structure of human disease-associated amyloid-forming proteins. Disease-associated amyloid proteins categorized into four types of native structures: (1) globular, (2) membrane or lipid-associated, (3) hormones, and (4) unknown or disordered. Many undergo proteolytic cleavage to produce fragments found in patient amyloid deposits (scissors). Amyloid proteins are each associated with neurodegenerative (red), systemic (blue), localized (green) or iatrogenic (orange) diseases.

Prior to conversion into disease-associated fibrils, amyloid-forming proteins exist in “native” states in their normal, physiological milieu. Although not all are globular, each native conformation is functional and thermodynamically favored. We categorized them into four native structure types: (1) globular, (2) membrane or lipid-associated, (3) hormones, and (4) unknown or disordered (Table 1, Figure 1). We propose that all amyloid-forming proteins can conform to Anfinsen’s hypothesis, even those that are “intrinsically disordered”.

Amyloid fibrils are most commonly formed by proteins with native globular structures, including β2-microglobulin, immunoglobulin heavy and light chains, transthyretin, and lysozyme. Their compact and soluble fold enable their function as enzymes, antibodies, and transport proteins. Interestingly, aggregation of many of these proteins is observed in systemic or localized amyloidoses. Amyloid fibrils also originate from membrane or lipid-associated proteins, which feature hydrophobic helices that remain in contact with lipids, such as apolipoproteins, or burial in membranes, such as amyloid-β precursor protein, integral membrane protein 2B, and transmembrane protein 106B. These proteins serve key roles as cell surface receptors, lipid transporters, and regulators of membrane adhesion. The third category of proteins is hormones, short signaling peptides mostly between 28 and 51 residues in length. Many polypeptides have intramolecular or inter-chain disulfide bonds that stabilize simple cyclic structures, such as those of atrial natriuretic peptide and somatostatin, or tertiary folds, such as that of insulin. Hormone proteins, as well as medicinal-turned-iatrogenic peptides also grouped into this category, are found in amyloid deposits localized to their organ of physiological activity or site of injection.

The fourth group of amyloid-forming proteins are those whose native structure is unknown or considered to be intrinsically disordered. This group includes tau, α-synuclein, and TDP-43, which are linked to the four most prevalent neurodegenerative disorders – Alzheimer’s disease, Parkinson’s disease, frontotemporal dementia, and amyotrophic lateral sclerosis. When studied *in vitro*, monomers of these brain amyloid proteins do not adopt compact tertiary folds but instead occupy an ensemble of conformations [8– 10], thus are deemed unstructured. Their structural malleability makes them suited for roles in transcriptional regulation and subcellular response [11]. However, intrinsically disordered proteins may not be fully disordered in their physiological milieu. First, these proteins have interactions that stabilize their conformations *in vivo*: tau binds and promotes assembly of microtubules, α-synuclein reversibly interacts with synaptic versicles (thus is also a membrane-associated protein), TDP-43 binds and regulates metabolism of RNA. Second, tau [12], α-synuclein [13], TDP-43 [14], and other proteins that aggregate in the brain [15-17] are capable of phase separation into condensates, often mediated by low complexity domains. While biomolecular condensates are highly dynamic and their organization is not fully understood [18], some *in vitro* evidence suggests intrinsically disordered proteins form ordered intermolecular bonds in RNA-protein granules and membraneless organelles, potentially featuring cross-β structure [19,20]. If one considers their native state to be in these condensates or in complex with binding partners, intrinsically disordered proteins are not as disordered *in vivo*, as they appear to be *in vitro*.

## Formation of amyloid fibrils

Whereas amyloid fibrils are unusually stable protein structures with deeper free energy minima than their native states, they take decades to form *in vivo* and in some cases, such as tau, are difficult to induce *in vitro*. The reason is nucleation of the amyloid scaffold presents the principal energetic barrier to amyloid formation.

**Figure 2.**
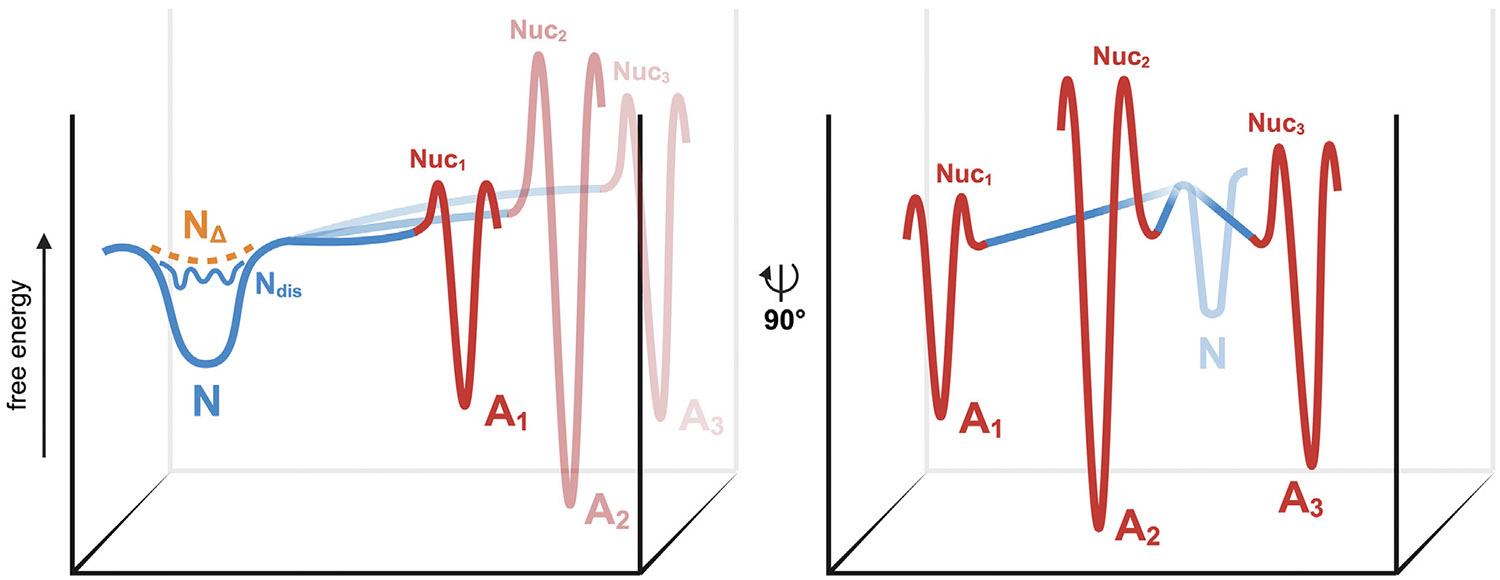
Schematic three-dimensional free energy landscape of amyloid-forming proteins. Amyloid-forming proteins have stable (N) or intrinsically disordered (N_dis_) native structures that pass through unequal barriers to amyloid nucleation (Nuc_1_, Nuc_2_, Nuc_3_) to form polymorphic amyloid fibrils (A_1_, A_2_, A_3_). Alteration of the amyloid-forming protein’s wildtype sequence by genetic mutation, alternative splicing, or proteolytic cleavage generates variant or fragment proteins (N_Δ_) higher in energy than the native state, reducing the barrier to amyloid formation.

Barriers to amyloid nucleation are represented by the peaks in free energy on the reaction pathways to the deep free energy wells of amyloid fibrils (Figure 2). The physical origin of these free energy peaks is the decrease of translational, rotational, and vibrational entropy of monomers required to establish a nucleus that specifies all unique intermolecular bonds that determine the fibril. Proteins carrying a high net charge present an enthalpic repulsion opposing formation of the amyloid nucleus. This repulsion can be reduced by oppositely charged cofactors or post-translational modifications (PTMs), thereby speeding fibril formation. Once the amyloid nucleus is present, addition of monomers to the fibril grants enthalpic release, leading to decrease in free energy, particularly at high monomer concentration. After some fibrils have formed, secondary nucleation [21], catalyzed on the surface of existing fibrils, may have a lower energy peak to climb than the initial nucleation.

Unequal barriers to amyloid nucleation may determine which amyloid polymorph prevails. In Figure 2, A1 would be the fastest growing fibril as it has the lowest free energy hill to climb before descent into its well, while A2 would be the slowest growing fibril as it has the highest free energy hill. Even though A2 is a more stable fibril structure, A1 may be the dominant fibril species due to its assembly efficiency, quickly sequestering monomers into its conformation. In this way, some argue that amyloid formation is kinetically controlled [22,23]. But as Anfinsen holds, a sequence forms the most stable structure accessible in its biological milieu, constrained by the energy states available in a system. In our hypothetical condition, the high energy of nucleation of A2 means it may not be accessible, whereas the less stable A1 structure with a lower nucleation barrier is accessible. If the physiological milieu changes (e.g. introduction of a cofactor that reduces the repulsion of the A2 structure), barriers to amyloid nucleation may change and another polymorph may be favored.

## Polymorphism of amyloid fibrils

An amyloid-forming protein has at least two free energy minimum conformations: its native state and its fibril state. Moreover, in its fibril state a given protein can adopt different fibril structures, a phenomenon known as polymorphism. Amyloid polymorphism underscores Anfinsen’s hypothesis that a protein’s sequence and its aqueous environment – “solvent, pH, ionic strength, presence of other components such as metal ions or prosthetic groups, temperature” – are both important determinants of structure [24].

**Figure 3.**
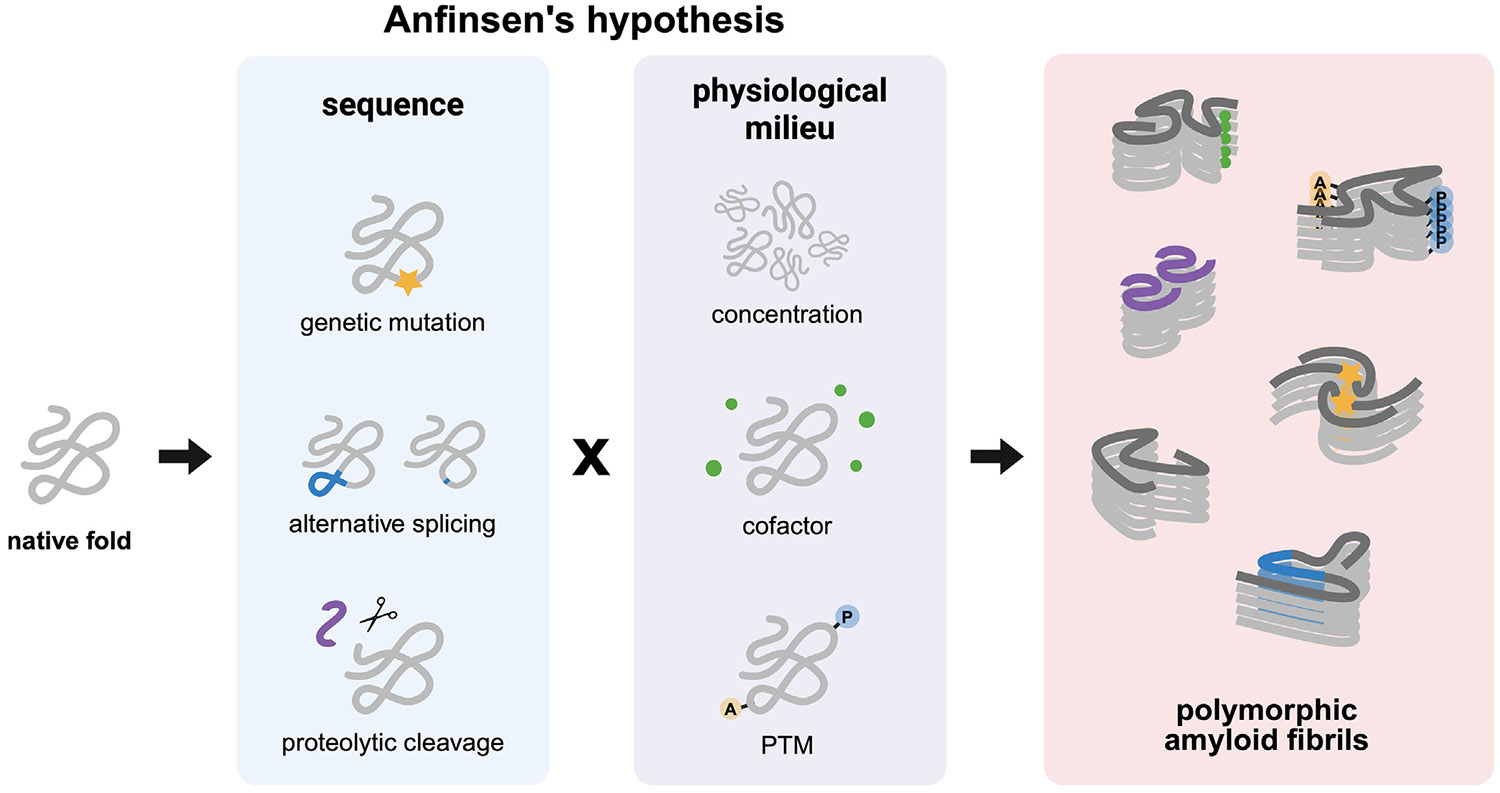
Anfinsen’s thermodynamic hypothesis for amyloid fibrils. Protein sequence and physiological milieu determine amyloid fibril structure *in vivo*.

Alteration of the sequence of an amyloid-forming protein can induce its aggregation and influence the fibril polymorph. Change to a protein’s wildtype sequence, evolved to fold or interact with binding partners, generates variant or fragment proteins that cannot access the native free energy minimum. These new protein molecules are likely disordered with unsatisfied hydrogen bonds, thus are in higher energy states with a reduced barrier to amyloid formation (Figure 2). Mechanisms that alter the wildtype sequence of amyloid-forming proteins include (1) genetic mutations, (2) alternative splicing, and (3) proteolytic cleavage (Figure 3). Disease-associated mutations, hereditary or sporadic, promote amyloid fibril formation through a variety of mechanisms summarized by Rosenberg [25], such as native structure destabilization, altered processing, subcellular mislocalization, and decreased binding to native partners. Point mutations can cause perturbations in amyloid structure, exemplified by the Arctic mutation E22G of amyloid-β [26] and the I84S mutation of transthyretin [27]. Alternative splicing affects the population of isoforms. In the case of tau, its ratio of proteins with three or four microtubule-binding repeat domains governs aggregation [28] and fibril fold [29]. Lastly, proteolysis by endogenous proteases often precedes, and sometimes is required for amyloid fibril formation. We annotated disease-associated amyloid-forming proteins that undergo proteolysis to produce fragments that are found in patient amyloid deposits (Table 1, Figure 1). Cleavage liberates fragments from the stable context of the full-length precursor, exposing amyloidogenic sequences normally protected in the native fold. This is especially common for globular and membrane-associated amyloid-forming proteins. Proteolysis is well characterized for some proteins, such as amyloid-β [30,31], while for other proteins, work remains to be done to elucidate processing pathways and participating enzymes.

A given amyloid-forming protein sequence can adopt various fibril structures, which are shaped by their distinct physiological milieu characterized by one or more factors. Amyloid polymorphism is well documented, especially of patient-extracted, *ex vivo* fibril structures of tau, amyloid-β, α-synuclein, and TDP-43 in neurodegenerative diseases. However in the same patient, and patients with the same disease, fibril structures are very similar, supporting the idea that amyloid fold defines molecular pathology [32]. (There are rare exceptions, such as multiple polymorphs of amyloid-b observed in of Alzheimer’s disease [33] and Down syndrome [34] cases.) The uniformity of fibril structure in one brain may be attributed to incubation in the same environment. The striking consistency of fibril structure in patients with the same diagnosis suggests that molecular characteristics that underlie each disease lead to particular polymorphs. In contrast to *ex vivo* amyloids, *in vitro* amyloids exhibit greater polymorphism, sometimes with many structures observed in one condition. Two recent cryo-EM timepoint studies captured the structural evolution of *in vitro* fibril formation of tau [35] and islet amyloid polypeptide [36], showing that they pass through intermediates before arriving to the mature fold.

## Physiological milieu of amyloid fibrils

Anfinsen wrote “in terms of natural selection… a protein molecule only makes stable, structural sense when it exists in conditions similar to those for which it was selected – the so-called physiological state”. Proteins fold into globular structures, evolved to be energetically favored in a healthy cellular environment. Similarly, amyloid fibrils make “stable, structural sense” in pathological contexts, that is their physiological milieu. Amyloid research has been investigating factors of the milieu which drive fibril formation in human diseases, including (1) protein concentration, (2) cofactors, and (3) PTMs (Figure 3).

Whereas a protein’s globular fold is favored at low concentration, high local concentration overcomes the entropy of nucleation and deepens the energy well of the amyloid state so that it becomes thermodynamically favorable. When solute concentration is maintained at a point exceeding its solubility limit (supersaturation), the solute protein is susceptible to aggregation [37]. Therefore, protein accumulation, by increased expression or decreased degradation, is a factor in amyloid formation. *In vitro* experiments showed that irreversible fibrils can arise from liquid-liquid phase separation of brain amyloid proteins [13-16,38]. This suggests dysregulation of protein-dense condensates may be a mechanism for amyloid nucleation *in vivo*.

PTMs alter the chemical property and size of protein molecules, and thus may promote aggregation and selection of fibril polymorphs. Pathological neuronal deposits of tau [39], α-synuclein [40], and TDP-43 [41] contain abnormally hyperphosphorylated forms of these proteins. Phosphorylation of tau compromises microtubule binding [42] and lowers its net positive charge to be more prone to self assembly. Proteomic studies of *ex vivo* tau species allow for comparison of the PTM profiles between tauopathies, which may reveal key modifications that yield diverse fibril structures [43,44]. This is supported by recapitulation of the Alzheimer’s disease fold *in vitro*, using recombinant tau proteins with mutations that mimic phosphorylation sites on Alzheimer’s disease tau [45].

Cofactors likely have a strong contribution to defining the physiological milieu of amyloid polymorphs. Patient-extracted, *ex vivo* fibrils are decorated by non-proteinaceous densities in cryoEM maps, which represent unidentified metabolites, metals or biological polymers consistently observed in the fibril structure. For tau, RNA is a prominent cofactor that colocalizes with deposits in tauopathies [46,47] and induces tau aggregation *in vitro* [48]. RNA’s negative phosphate backbone interacts with positive lysine residues of tau, not only compensating charge but potentially also guiding it to the final conformation [49]. For α-synuclein, lipids colocalize in Lewy bodies of Parkinson’s disease [50]. Fibril complexes formed *in vitro* show acyl chains of lipids interacting with hydrophobic residues of α-synuclein, mediating alternative polymorphs [51]. In one disease known so far, another protein is the essential cofactor for fibril formation. Amyloid fibrils in frontotemporal lobar degeneration with TDP-43 inclusions (FTLD-TDP) type C patients feature a heteromeric amyloid core consisting of TDP-43 and annexin A11 [52].

*In vitro* experiments are helpful to isolate individual variables to test their effects, to reveal biophysical principles underlying amyloid formation. At what critical concentration does an amyloid protein convert from liquid-liquid phase separation to irreversible fibrils? Which cofactor or PTM induces a particular disease-associated amyloid fold? More complex models, such as stem cells differentiated to specific cell types and 3-dimensional organoids, are used to tease apart cellular programming and pathways implicated in protein aggregation. As *in vitro* systems are applied to test hypotheses, the challenge is discerning what is causative and what is a manifestation of the disease milieu.

## Conclusion

Despite the complexity of biological tissues and our still incomplete knowledge of the repertoire of molecular constituents and their concentrations, Anfinsen’s concept of the *in vivo*, physiological milieu as a determinant of protein structure, when coupled with the laws of thermodynamics, remains a guiding principle of protein science. Even the challenge presented by more recent discoveries of the amyloid state of proteins, such as polymorphic structures accessible to a given amino acid sequence, can be understood through the hypothesis he put forward 70 years ago.

## Figures and Tables

**Table 1:** Native structure of human disease-associated amyloid-forming proteins. Adapted from Amyloid Nomenclature 2022 [53] and Chiti and Dobson [54]. In addition to the 42 proteins from Amyloid Nomenclature, we included 3 more with convincing evidence of their amyloid aggregation in human disease: huntingtin [55], TDP-43 [56,57] and TAF15 [58].

Protein	Native structure	Proteolytic Fragment	Disease
α-Synuclein	Disordered, membrane or lipid-associated	C-terminal truncated proteins deposit in Lewy bodies [59]	Neurodegenerative
Amyloid-β precursor protein	Membrane or lipid-associated	Amyloid-β produced by cleavage by β- and γ-secretases [30,31]	Neurodegenerative
Apolipoprotein A I	Membrane or lipid-associated	N-terminal fragment found in amyloidosis of various organs [60]	Systemic
Apolipoprotein A II	Membrane or lipid-associated	C-terminal fragment of 21 residue extension caused by stop codon mutation found in renal amyloidosis [61]	Systemic
Apolipoprotein A IV	Membrane or lipid-associated	N-terminal fragment found in amyloidosis of the heart and kidney [62]	Systemic
Apolipoprotein C II	Membrane or lipid-associated		Systemic
Apolipoprotein C III	Membrane or lipid-associated		Systemic
Atrial natriuretic factor	Hormone		Localized
β2-microglobulin	Globular	N-terminal truncated proteins detected in haemodialysis patient [63]	Systemic
Calcitonin	Hormone		Localized
Cathepsin K	Globular	C-terminal fragment in amyloid component of a kidney angiomyolipoma [64]	Localized
Corneodesmosin	Unknown		Localized
Cystatin C	Globular	Truncated N-terminus aggregates in hereditary cerebral hemorrhage with amyloidosis [65]	Systemic
EGF-containing fibulin-like extracellular matrix protein 1 (EFEMP1)	Membrane or lipid-associated	C-terminal fragment in venous amyloid deposits from large intestine of elderly patients [66]	Localized
Enfuvirtide	Peptide		Iatrogenic
Fibrinogen α chain	Globular	Amyloid consists of C-terminal fragment found in hereditary renal amyloidosis [67]	Systemic
Gelsolin	Globular	Furin cleavage produces amyloid fragments [68,69]	Systemic
Glucagon	Hormone		Localized
Glucagon-like peptide-1	Hormone		Iatrogenic
Huntingtin	Globular	N-terminal fragments of polyglutamine repeats aggregate in Huntington’s disease [70]	Neurodegenerative
Immunoglobulin heavy chain	Globular	Amyloid fibrils composed of fragments from the variable region [71]	Systemic
Immunoglobulin light chain	Globular	Amyloid component contains fragments from the variable region [72]	Systemic
Insulin	Hormone		Iatrogenic
Integral membrane protein 2B	Membrane or lipid-associated	Furin cleavage generates ABri and ADan peptides, which deposit in familial British dementia [73] and familial Danish dementia [74] patients, respectively	Neurodegenerative
Interleukin-1 receptor antagonist	Globular		Iatrogenic
Islet amyloid polypeptide	Hormone		Localized
Lactadherin	Membrane or lipid-associated	A 50 residue fragment, known as medin, accumulates in aortic medial amyloidosis [75]	Localized
Lactotransferrin	Globular		Localized
Leukocyte cell-derived chemotaxin 2 (LECT2)	Globular		Systemic
Lysozyme	Globular		Systemic
Odontogenic ameloblast-associated protein	Unknown	N-terminal fragment in amyloids of calcifying epithelial odontogenic tumors [76]	Localized
Parathyroid hormone	Hormone		Localized
Prion protein	Membrane or lipid-associated	Proteolytic processing generates amyloidogenic fragments in Gerstmann-Sträussler-Scheinker [77] and Creutzfeldt-Jakob disease [78]	Neurodegenerative
Prolactin	Globular	N-terminal fragment aggregates in pituitary adenoma [79,80]	Localized
Semenogelin-1	Unknown	N-terminal fragment found in senile seminal vesicle amyloid [81]	Localized
Serum amyloid A	Membrane or lipid-associated	N-terminal fragment in fibrils [82,83]	Systemic
Somatostatin	Hormone	Processed peptides found in amyloid deposits of neuroendocrine tumors [84,85]	Localized
Surfactant protein C	Membrane or lipid-associated		Localized
TAR DNA-binding protein 43 (TDP-43)	Disordered	C-terminal fragments aggregate in ALS and FTLD [41,86]	Neurodegenerative
TATA-binding protein-associated factor 15 (TAF15)	Disordered		Neurodegenerative
Tau	Disordered	Glu391 [87] and Asp421 [88] truncated tau detected in Alzheimer’s disease	Neurodegenerative
Transforming growth factor β induced protein (TGFBIp)	Globular	C-terminal fragments, potentially cleaved by HtrA1 protease, enriched in corneal amyloid deposits [89]	Localized
Transmembrane protein 106B (TMEM106B)	Membrane or lipid-associated	Luminal β immunoglobulin-like domain cleaved prior to amyloid formation [90-92]	Neurodegenerative
Transthyretin	Globular	Deposits with C-terminal fragment have distinct pathology compared to deposits with full-length protein [93]	Systemic

## Data Availability

No data was used for the research described in the article.
